# Radiation for hematologic malignancies: from cell killing to immune cell priming

**DOI:** 10.3389/fonc.2023.1205836

**Published:** 2023-06-13

**Authors:** Bouthaina Dabaja, Michael Spiotto

**Affiliations:** Department of Radiation Oncology, The University of Texas MD Anderson Cancer Center, Houston, TX, United States

**Keywords:** radiation, immunotherapy, cell therapy, immunogenic, bridging

## Abstract

Over the past half-century, the role of radiotherapy has been revolutionized, in part, by a shift from intent to directly kill cancer cells to the goal of priming anti-tumor immune responses that attack both irradiated and non-irradiated tumors. Stimulation of anti-tumor immunity depends on the interplay between radiation, the tumor microenvironment, and the host immune system, which is a burgeoning concept in cancer immunology. While the interplay of radiotherapy and the immune system has been primarily studied in solid tumors, we are beginning to understand this interplay in hematological malignancies. The intent of this review is to lead readers through some of the important recent advances in immunotherapy and adoptive cell therapy, highlighting the best available evidence in support of incorporating radiation therapy and immunotherapy into the treatment of hematological malignancies. Evidence is presented regarding how radiation therapy ‘converses’ with the immune system to stimulate and enhance anti-tumor immune responses. This pro-immunogenic role of radiotherapy can be combined with monoclonal antibodies, cytokines and/or other immunostimulatory agents to enhance the regression of hematological malignancies. Furthermore, we will discuss how radiotherapy facilitates the effectiveness of cellular immunotherapies by acting as a “bridge” that facilitated CAR T cell engraftment and activity. These initial studies suggest radiotherapy may help catalyze a shift from using chemotherapy-intensive treatment to treatment that is “chemo-free” by combining with immunotherapy to target both the radiated and non-irradiated disease sites. This “journey” has opened the door for novel uses of radiotherapy in hematological malignancies due to its ability to prime anti-tumor immune responses which can augment immunotherapy and adoptive cell-based therapy.

## 1 Beyond cell killing: the systemic effects of radiation therapy

The dawn of immunotherapy for cancer began with the realization that hematologic cancer both originates from and interacts with components of the immune system. The success of therapy involving antibodies such as rituximab targeting the B cell specific marker CD20 for hematologic cancers prompted an explosion of interest in immune-based therapy. A parallel development over the past few decades has focused on ‘de-escalating’ treatment in attempts to maintain oncologic control while minimizing the toxicity of classic anticancer therapy. For hematologic malignancies, this approach has meant shifting from aggressive chemotherapy to combinations of chemotherapy and immunotherapy to completely chemo-free strategies, such as using adoptive cell therapy as salvage for the disease that has relapsed. Similarly, the use of checkpoint inhibitors such as nivolumab and pembrolizumab has resulted in 65-87% response rates in previously treated Hodgkin’s Lymphoma (1, 2). Advances in the understanding of immune functions have led to cancer immunotherapy strategies that exploit different mechanisms of action, from activating innate and adaptive immune effector mechanisms to blocking inhibitory and suppressive mechanisms (3, 4).

**Figure 1:**
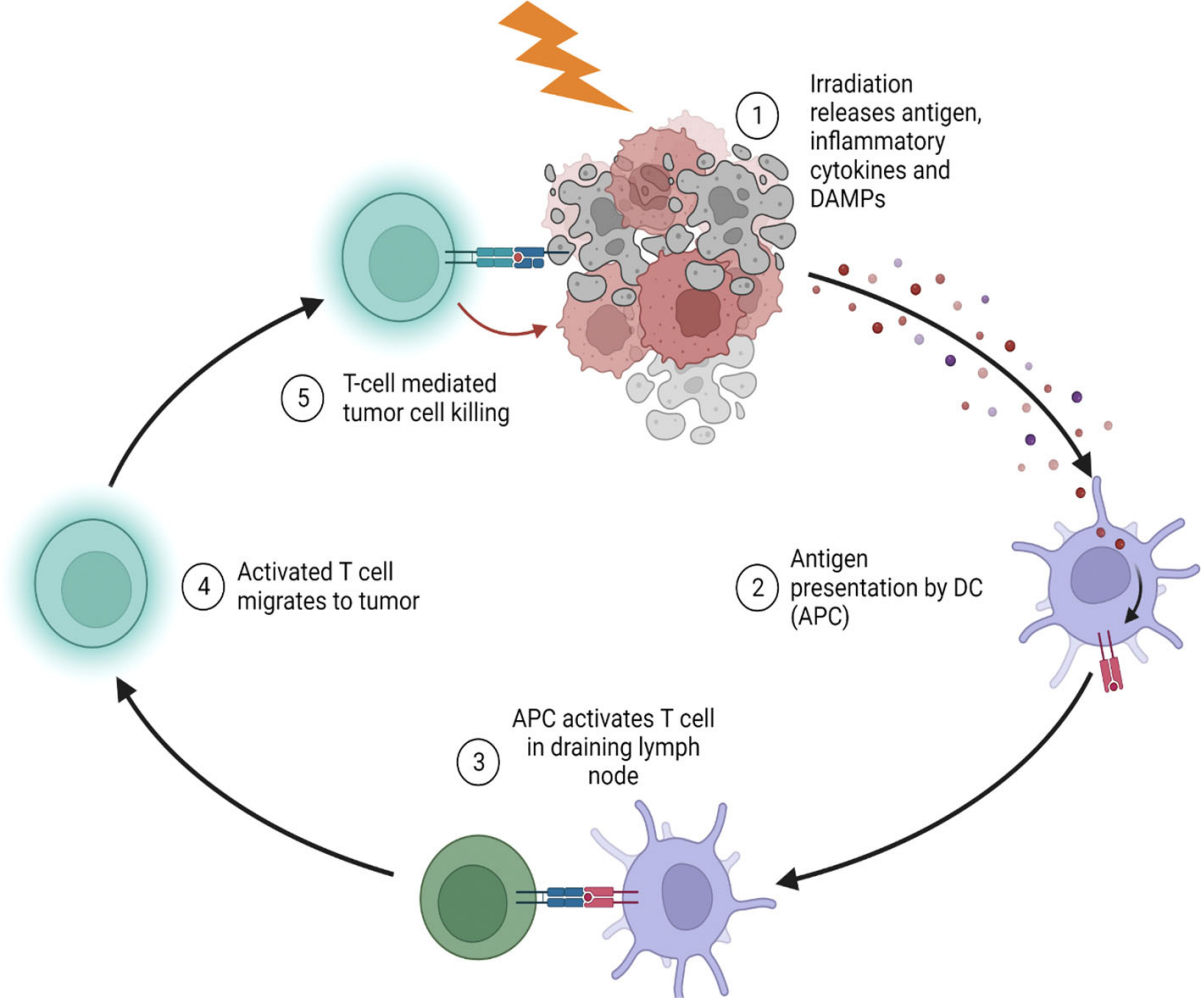
Radiation stimulates tumor antigen release and stimulate anti-tumor immunity. (1) Radiotherapy causes immunogenic cell death with the release of damage associated molecular pattern proteins, inflammatory cytokines and antigen. (2) Antigen presenting cells (APC) within the tumor phagocytose antigens and mature due to the inflammatory environment in the irradiated tumor. (3) Mature APCs migrate to the draining lymph node to activate T cells which then migrate to the irradiated tumor (4). (5) Activated tumor infiltrating T cells further cause destruction of tumors *via* direct cell killing or by releasing cytotoxic cytokines.

Radiation therapy has undergone a similar transformation, with a reduction of radiation fields and doses motivated by the urge to avoid long-term toxicity that increases the morbidity and mortality of long-term survivors. Evidence that low-dose radiation contributes to higher remission rates, along with preclinical findings indicating that radiation can evoke immune-mediated antitumor effects, led to the proposition of focal, low-dose radiotherapy regimens may act as an immune modulator. The immune modulatory effects of radiation begin with the radiation-induced release of tumor antigens from cancer cells coincident with dendritic and antigen-presenting cell maturation to enhance T-cell priming (**Figure 1**). Furthermore, radiotherapy may alter local immunosuppressive cells such as regulatory T cells, myeloid-derived suppressor cells, and macrophages to enhance T cell killing of irradiated tumor cells. Evidence that radiation therapy can augment both the adaptive and innate immune system is one of the most significant advances that change how we think about radiation therapy as having both local and systemic effects.

### 1.1 Interplay between radiation and the immune system

Awareness that the effectiveness of radiation therapy depends on an intact immune system began as early as the late 1970s when clinicians observed that more severe lymphopenic states were associated with worse outcomes in multiple cancer types after radiotherapy. At that time, preclinical experiments in controlled *in vivo* settings were enlightening, but the failure to translate the findings to the clinic was equally enlightening. Subsequently, Lee et al. found that radiation could eradicate B16 melanoma tumors in an immunocompetent mouse model, but not in T cell–deficient nude mice (5). Thus, pre-clinical and clinical data suggest that a functional immune system was critical for the control of cancer by radiotherapy.

Even though the immune system is vital to the effectiveness of radiotherapy, several sophisticated processes underlie this mini-cold war” between the immune system and cancer cells entrenched in the tumor microenvironment. Tumors evade immune responses and progress by eliciting immunosuppressive mechanisms to prevent immune destruction. Classically, immune rejection of tumors requires the activation of CD8^+^ tumor-specific cytotoxic T cells that directly kill the cancer cells. To a lesser extent, macrophages, NK cells, and B cell/antibody responses may also play a role in inhibiting tumor growth. However, the depletion of CD8^+^ T cells consistently reduced the efficacy of radiotherapy in multiple preclinical models. These active CD8^+^ T cells are inhibited by various suppressive cells including CD4^+^ regulatory T cells (Tregs), myeloid-derived suppressor cells (MDSCs), macrophages, and by soluble factors such as TGFβ. Of note, Tregs’ primary role is to inhibit cytotoxic T-cell activity (6, 7).

Localized radiotherapy initiates cancer cell death by promoting the secretion of immunogenic cell death markers, cytokines, and chemokines in the tumor microenvironment that leads to the maturation of dendritic cells, and macrophages to activate tumor specific CD8^+^ T cells, which then infiltrate the tumor (8, 9). Radiation also releases antigens from dying cancer cells to facilitate the cross-presentation of tumor antigens, along with MHC class II molecules, by dendritic cells. Moreover, radiation can induce the expression of chemokines CXCL-9, -10, -11, and -16, which leads to the recruitment and chemotaxis of T cells to the tumor microenvironment (10, 11).

Radiation can cause paradoxical immune effects. While activating adaptive and innate immune responses as described above, radiation can also stimulate the differentiation of Tregs, *via* TGF-β and interleukin (IL)-10, which inhibits activated T-cells to promote tumor progression (12–14). Radiation can have divergent effects on CD4^+^ Tregs, which are characterized by the expression of the forkhead box transcription factor (Foxp3). Beauford et al. demonstrated that both natural and TGF-β induced CD4^+^ Tregs were more resistant to radiotherapy than conventional CD4^+^ T cells isolated from human peripheral blood samples (15). However, radiation also caused decreased suppressive activity and downregulation of Foxp3, especially in TGF-b-induced Tregs. Other groups using a lower radiation dose of less than 2 Gy caused more Tregs depletion compared to the number of conventional CD4^+^ T cells (16). Consequently, there is a complex interplay between anti-tumor immune responses and immunosuppressive cells in irradiated cancers that may further complicate immunotherapy approaches.

To summarize, radiation therapy holds promise for its ability to positively modulate the immune system, but as discussed further below, additional clinical and preclinical studies are needed to determine the ideal radiation dose, timing, and sequence when radiation is used with other forms of therapy.

### 1.2 The rationale for combining radiation with immunotherapy

Since radiation causes paradoxical effects on anti-tumor immune responses, much attention is focused on optimizing the combination of radiotherapy with immunotherapy, particularly with regard to the timing of the two therapies. Optimizing radiotherapy with immunotherapy will synergistically influence the antitumor immune response by maintaining its antitumor effects while limiting the immunosuppressive effects of radiation.

Strategies to reduce immunosuppressive mechanisms have included combining radiation (1) with cyclophosphamide, which induces antitumor immune responses by enhancing the differentiation of T helper 17 cells and/or depletion of Tregs (17); (2) with neutralizing antibodies which target CD25 expressed by regulatory T cells; and/or (3) with immune checkpoint molecules such as cytotoxic T lymphocyte-associated protein 4 (CTLA4) and programmed cell death (PD1) (18–20).

A group from the University of Pennsylvania (21) elegantly demonstrated the importance of timing and sequence in combining radiation with anti-CTLA4 or anti-PD1 immunotherapy for the treatment of melanoma. They also explored the mechanism by which resistance to this therapy develops. This group found that combining anti-CTLA4 antibodies with radiation produced an initial response, but resistance was both common and developed quickly. Analyses with mouse models showed that the resistance was due to the upregulation of PDL1 on the melanoma cells and was associated with T-cell exhaustion. Notably, adding PDL1 blockade reversed the T-cell exhaustion and mitigated the depression in the ratio of CD8^+^ cells to Tregs. Others have also found that radiotherapy-induced antitumor immunity contributes to the therapeutic efficacy of the radiation and can be augmented by CTLA4 blockade (22). A third group found that resistance to radiation in a mouse mode of colorectal cancer could be overcome by the use of radiation first followed by IL-12 (23). These studies and others emphasize that the sequence of combining radiotherapy with immunotherapy as well as the type of immunotherapy used can drastically alter the efficacy of the combination therapy.

**Figure 2:**
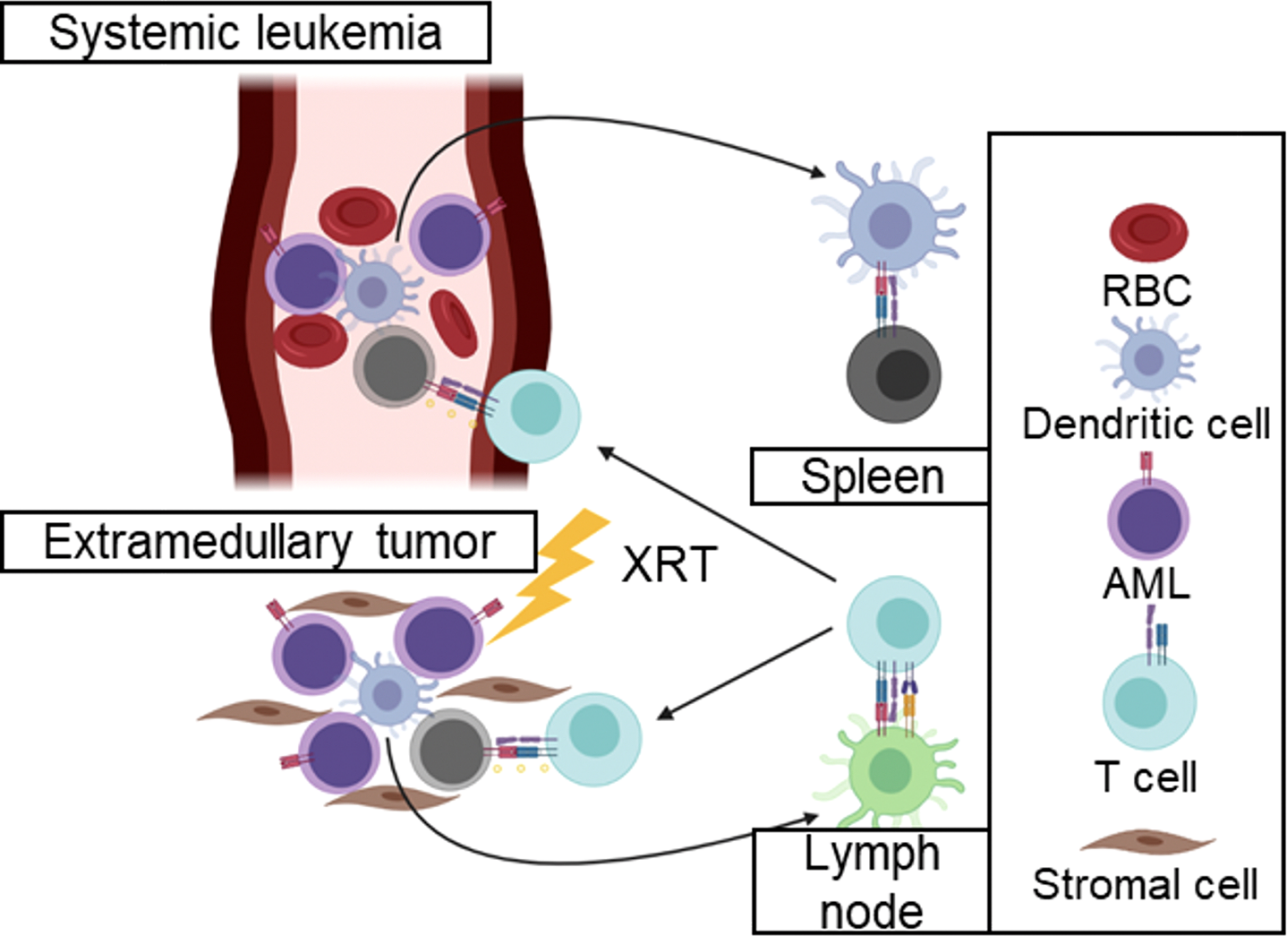
Irradiation of hematological cancers may activate immune responses against systemic disease. Irradiation of extramedullary tumors may stimulate immune responses in the lymph node that would otherwise be tolerated in the spleen. These immune responses may clear circulating leukemia cells without the local immunosuppressive mechanisms that inhibit anti-tumor immune responses in solid tumors.

Furthermore, the irradiation of hematological cancers that present in extramedullary sites may stimulate immunity toward systemic disease. The Kline group has demonstrated that hematological cancers induce states of T-cell tolerance in the spleen (24, 25). By contrast, it is likely to radiotherapy of the extramedullary disease may potentiate immune responses against circulating cancer cells that are not subject to immunosuppressive microenvironments (**Figure 2**). Consequently, there remains an unmet need to incorporate radiotherapy with immunotherapy for hematological cancers.

A large body of clinical literature is available on the use of radioimmunotherapy for solid and liquid cancers. In most of these trials, radiation is combined with ipilimumab, which targets CTLA4 (NCT01935921, NCT02254772, NCT01497808). Studies of radioimmunotherapy for hematologic malignancies are scarcer than those for solid tumors, primarily because of the success of systemic therapy for the newly diagnosed disease, especially when radiation is used for consolidation, and because radiation is rarely combined with immunotherapies for salvage regimens or aggressive hematologic malignancies. This is a young yet promising field that is making steady progress.

Low-dose radiation has been successfully used as a curative modality for various hematologic cancers, including marginal zone lymphoma, mantle cell lymphoma, mycosis fungoides, and myeloma (26–29). The success of this less toxic treatment has led some groups to consider combining low-dose radiation with immunotherapy. Hammerich et al. combined low-dose radiotherapy with Flt3 ligand and a Toll-like receptor 3 agonists in indolent lymphomas. Here, they observed regression in 8/11 irradiated tumors. Furthermore,6 of 11 patients had regression in non-irradiated tumors (i.e. abscopal effects) and 3 patients had a complete or near-complete response at all disease sites (30). Another example being tested in NCT04054167 explores the use of ultra-low-dose radiation with chemotherapy-free targeted therapy for relapsed or refractory mantle cell lymphoma. In another trial (NCT05357794), total skin electron-beam radiation therapy is being tested with concurrent brentuximab vedotin, an anti-CD30^+^ agent, for stage I-IV mycosis fungoides/Sezary syndrome. This trial includes analysis of immune biomarkers and their contribution to clinical responses. These trials, and others currently being proposed, underscore the need for “granular” data regarding changes in the immune system in response to radiation alone or radiation combined with immunotherapy.

## 2 Radiation with adoptive cell therapy

Another form of immunotherapy under intense study is adoptive cell therapy, which is often—but not always—based on T cells. T-cell killing is mediated by T-cell receptors (TCR) on antigen-specific T cells that bind tumor-antigen peptides presented by major histocompatibility complex (MHC) molecules in mice or Human Leukocyte Antigen (HLA) in humans on the surface of cancer cells. Thus, the success of such therapy depends not only on antigen expression by cancer cells but also on the sustained mobilization of sufficient numbers of effector T cells to eliminate the tumor.

Early forms of adoptive cell therapy were based on donor lymphocyte infusion, which involved extracting lymphocytes from donated blood or tissue samples, followed by sorting and expansion of cytotoxic T cells *ex vivo*, and infusion of those cells into the patient. The success of this technique (the basis for allogeneic stem cell transplantation) was tempered by the challenge of graft-versus-host disease, caused when the donor T cells (graft) provoked an immune reaction against the host. Attempts to reduce the severity of graft-versus-host disease led to strategies such as depleting T cells from the donor graft (31); unfortunately, that strategy led to higher rates of disease relapse, infection, and graft failure (32). Donor lymphocyte infusion can also be repeated after an initial graft to reduce infections, but its primary purpose is to help increase antitumor immune surveillance and prevent relapse (33).

The first forms of adoptive cell therapy were done with tumor-infiltrating lymphocytes, that is, autologous lymphocytes that had been expanded *ex vivo* with IL-2. Reinfusion of these cells often produced clinical responses, but the limited survival of the T cells and subsequent “escape” by the tumor led to relapse. The natural next step in the evolution of adoptive cell therapy was to genetically engineer T cells to overcome the shortcomings associated with tumor-infiltrating lymphocytes. This genetic engineering was the basis for the most significant advances in treating hematologic malignancies to date, using what is now referred to as chimeric antigen receptor (CAR) T-cell therapy.

### 2.1 CAR T-cell therapy

The initial premise of CAR T-cell therapy was to engineer T cells by incorporating genes encoding artificial TCR-like molecules formed by a single chain variable antibody fragment (ScFv), spacers, transmembrane domains, and intracellular signaling components to facilitate the recognition of tumor-specific antigens. The T cells can come from either the patient or from donor-derived immune cells, and the engineering leads them to express recombinant or chimeric antigen receptors on their surface. Eventually, the T cells, recognize a target-specific tumor-associated antigen and lead to an immune-induced mediated attack that leads to tumor cell death. Manufacturing CAR T cells by T-cell genetic engineering takes place in the following steps: apheresis; T-cell enrichment; gene modification, activation, and *ex vivo* expansion; and reinfusion to the patient.

The first form of CAR T-cell therapy to be approved by the US Food and Drug Administration (FDA) was tisagenlecleucel, which was approved in August 2017 for relapsed/refractory acute lymphoblastic lymphoma in pediatric patients and young adults. The next CAR T-cell therapy to be approved (in October 2017) was the CD19-targeted axicabtagene ciloleucel (also known as axi-cel), which had been shown in the international ZUMA-1 trial to lead to high rates of complete remission in patients with relapsed refractory large B cell lymphoma. Lisocabtagene Ciloleucel is a third anti-CD19 CAR-T cell approved for relapsed and refractory large B-cell lymphoma with an objective response rate of 73% and complete response rate of 53% (34, 35). The US FDA has subsequently approved other CAR T-cell products that generally differ from each other according to the costimulatory domain used for T-cell activation or intracellular signaling (36–42).

Notably, CAR T-cell therapy is associated with significant toxicity, including cytokine release syndrome, neurotoxicity, and, in rare cases, capillary leak syndrome and multiorgan failure. Thus, patients undergoing this type of therapy generally are continuously monitored in an inpatient hospital setting (43, 44).

### 2.2 Challenges with CAR T-cell therapy: a role for radiation?

CAR T-cell therapy has significantly improved the management of relapsed or refractory hematologic malignancies; however, to date, durable remissions have been achieved in less than half of treated patients (45). Strategies underway to improve CAR T-cell efficacy include improving the specificity and efficacy of the target; reinvigorating exhausted T cells; overcoming Treg- and myeloid cell–mediated immunosuppression; promoting CD8^+^ cell activity; and increasing myeloid cell recruitment and antigen presentation. Other approaches include seeking new target antigens for broader use of adoptive T-cell therapy, including the use of NK cells modified with an anti-CD19 CAR. (The benefit of NK cells is that they carry little to no associated risk of cytokine release syndrome, neurotoxicity, or graft versus host disease) (46). Another consideration, as discussed further below, is the preparative regimen before the transfer of the adoptive cells. These regimens currently involve lymphodepletion conditioning by various means to make space for the incoming cells. Lymphodepletion is thought to work by eliminating “sinks” of homeostatic cytokines such as IL-2, IL-7, and IL-15, eradicating immunosuppressive regulatory cells and myeloid-derived suppressors, and promoting the expansion, function, and persistence of the transferred cells. In one example, lymphodepletion with fludarabine and cyclophosphamide led to increased serum IL-15 levels and was associated with improved outcomes after CAR T-cell therapy (47). Lymphodepletion also increased levels of stimulatory cytokines such as IFN-γ, which facilitate CAR T-cell trafficking (48–50). Radiation is also an effective means of lymphodepletion in addition to a means of stimulating tumor-specific immunity *via* increasing tumor cytotoxic lymphocytes through tumor antigen presentation and MHC 1 expression.

#### 2.2.1 Radiation as “bridge therapy” before adoptive cell therapy

The use of radiation therapy as a “bridging” strategy before adoptive cell therapy was initially intended as a way to “buy time” for patients with rapidly progressing diseases while they await cell manufacturing. Clinical studies have shown that the use of sublethal radiation doses is safe and can improve outcomes in patients undergoing CAR T-cell therapy, especially those with adverse factors such as bulky disease. Sim and colleagues reported that giving radiation, to a median dose of 20 Gy in 2- to 4-Gy fractions, to 12 patients with poor-prognosis diffuse large B-cell lymphoma as a bridge before axi-cel therapy led to an overall response rate of 82%, and 5 of 11 patients (45%) achieved a complete response (51). A group from MD Anderson Cancer Center retrospectively evaluated the effects of radiation as bridge therapy for 148 patients with relapsed or refractory large B-cell lymphoma (52) and found that the 1-year progression-free survival rate for patients who received any bridging therapy was 29%, compared with 44% for patients who did not (P=0.06). Furthermore, among 45/124 patients receiving bridging therapy before CAR-T infusion, RT bridge was better compared to systemic bridging therapy in terms of objective response rate (100% vs. 67%) and complete response rates (82% vs. 38%). The benefit of RT bridging was further improved by comprehensively treating all disease sites rather than focally treated select sites of disease. Notably, the 62 patients who received bridge therapy were more likely to have poor prognostic features at the time of apheresis. Other retrospective series from the University of Pennsylvania and Memorial Sloan Kettering Cancer Center have shown that radiation used as bridge therapy can have other beneficial effects in addition to lymphodepletion before CAR T-cell therapy, including palliation and focal cytoreduction, which can maximize the number of patients who reach the infusion stage without increasing toxicity (53, 54). We typically deliver radiation therapy after pheresis and before CAR-T cell infusion. We are flexible with the radiation regimen and number of days to accommodate the CAR T cell manufacturing time, however, we try to give an average dose of 20 Gy.

Another study of 96 patients with large B-cell lymphoma treated with commercially available axi-cel at Moffitt Cancer Center identified high tumor burden (assessed by PET-CT) as being associated with significantly shorter progression-free and overall survival (55). These and other studies support the notion that optimal tumor debulking can improve outcomes after CAR T-cell therapy (56, 57). Thus, the use of radiation therapy can overcome some adverse factors and improve outcomes (51–54).

#### 2.2.2 Other considerations for combining CAR T-Cell therapy with radiation

##### 2.2.2.1 T-cell fitness

The safe introduction of radiation in combination with CAR T-cell therapy requires understanding the complexity of adoptive cell therapy and how it is (or could be) affected by prior or concurrent therapy. The polyfunctionality or “fitness” of CAR T cells affects both responses to treatment and toxicity (50). Patients who respond to CAR T-cell therapy tend to have higher percentages of effector T cells than do non-responders, and superior clinical responders have higher levels of memory CD8+ T cells. Therapies that are likely to cause prolonged cytopenia, particularly in patients who are older or less fit, could have a greater negative effect on T-cell fitness as well as on patient outcomes. For instance, additional cycles of chemotherapy in patients with acute lymphoblastic leukemia, non-Hodgkin lymphoma, Hodgkin lymphoma, or acute myelogenous leukemia led to the depletion of naïve effector memory T cells and reduced T-cell proliferation (58). CAR T-cell fitness also varies by the number of prior lines of therapy received; median peak CAR T-cell levels and median CAR T-cell expansion levels were greater in the ZUMA-12 patient cohort than in the ZUMA-1 cohort (59). These observations suggest that exposure to multiple oncologic therapies can adversely affect the function of autologous cells used for CAR T-cell production. Bendamustine in particular can adversely affect T-cell numbers and function (60, 61).

##### 2.2.2.2 Radiation timing

Although the optimal timing for radiation in the CAR T-cell setting has not been well studied, the radiosensitivity of lymphocytes would suggest that radiation therapy be introduced after apheresis to avoid reducing lymphocyte counts and impairing cell collection (62, 63). We generally recommend that radiation be given after apheresis, for this reason, to avoid affecting circulating T cells before the apheresis. Given what we know about radiation’s positive effect on the antigen-presenting mechanism, and its ability to increase T-cell effectors, one might speculate that CAR T cells should be infused no more than 2-5 days after the conclusion of radiation. However, some evidence is emerging to suggest that local radiation could also be beneficial for priming the immune system in favor of CAR T-cell therapy. For example, Young et al. showed that varying the sequencing of triple-combination therapy with anti-CTLA4, radiation, and anti-OX40 had diverging antitumor efficacy in a preclinical mouse model of breast cancer, perhaps because the timing affected the mechanism of action for each component of the immunotherapy (64). The assumption of synergy between treatment modalities is also supported by a case of a patient with relapsed/refractory multiple myeloma given B-cell maturation antigen (BCMA)-targeted CAR T-cell therapy followed by radiation therapy for spinal cord compression. This combination led to a cytokine-release syndrome, including a peak in TCR repertoire expansion and increased serum IL-6 and C-reactive protein (CRP), which took place later than expected for CAR T-cell therapy alone (65). The patient showed a complete systemic response, including persistent BCMA CAR T cells, raising the intriguing possibility that radiation may influence both the local and distant treatment response. Finally, a group at Memorial Sloan Kettering Cancer Center reviewed 14 patients who had received salvage radiation for non-Hodgkin lymphoma that had progressed after CAR T cell therapy (53). These patients experienced a remarkably long median overall survival time of 10 months after the salvage radiation; 6 patients with localized relapse had a response rate of 100%, 3 of whom were “bridged” to allogeneic transplantation; and all 3 patients were alive without evidence of disease at the time of the analysis.

##### 2.2.2.3 Radiation target and dose

The optimal radiation dose and target are not yet determined for CAR cell therapy. Although some published data suggest that high radiation doses and comprehensive treatment of all affected sites could be beneficial (52, 54), those data were based on retrospective analyses in which comprehensive-site radiation treatment was done only when all sites could be encompassed within the radiation field. As for the dose, patients who did better with higher radiation doses could be those who had been expected to undergo a long course of radiation while waiting for CAR T cell manufacturing, i.e., patients without rapidly progressing disease. Although the optimal dose of radiation in combination with CAR T-cell therapy is under investigation, early preclinical and clinical evidence suggests that a rapid (hypofractionated) course of radiation can avoid lymphopenia and also result in the recruitment of dendritic cells, priming of anti-tumor CD8^+^ T cells, and a relatively low number of infiltrating regulatory T cells. These findings may serve as an early rationale for considering hypofractionated radiation schedules over conventionally fractionated schedules (66, 67).

In summary, radiation should be delivered after apheresis, if possible, to minimize its effects on T-cell fitness, although more comprehensive radiation treatment may be helpful if it can be delivered safely with minimal toxicity. Hypofractionated regimens may result in a more favorable immune microenvironment and minimize toxicity that may require treatment with steroids.

## 3 Summary and future directions

Advances in cancer immunotherapy, especially recent advances in cell-based therapies, are heralding unprecedented successes in the treatment of hematologic malignancies; the addition of radiation therapy to immunotherapy could further strengthen this approach through radiation’s well-studied ability to activate the immune system that subsequently recognizes and kills malignant cells. Advances in cancer immunotherapy have been possible thanks to developments in cancer genomics and biology, which give us “high-resolution” insights as to what happens at the cellular and molecular levels as cancer develops, evolves, and takes over. Therefore, researchers must recognize that the future of cancer research requires making the leap from looking at clinical markers in clinical studies to looking at cellular and molecular markers to further advance cancer therapy. Translational studies are needed to clarify the mechanisms by which cancer evades the immune system if we are to effectively and appropriately apply immunotherapy, adoptive cell therapy, and radiation therapy, singly or in various combinations, in the most effective and least toxic way possible.

## Author contributions

Both authors contributed to the writing and editing of the manuscript.
